# Absence of antibodies to *Rickettsia spp., Bartonella spp., Ehrlichia spp. and Coxiella burnetii* in Tahiti, French Polynesia

**DOI:** 10.1186/1471-2334-14-255

**Published:** 2014-05-12

**Authors:** Didier Musso, Julien Broult, Philippe Parola, Didier Raoult, Pierre-Edouard Fournier

**Affiliations:** 1 Pôle de recherche et de veille sur les maladies infectieuses émergentes, Institut Louis Malardé, Tahiti, PO Box 30, 98713 Tahiti, Polynésie française; 2 Centre de transfusion sanguine de la Polynésie française, PO Box 4530, 98713 Tahiti, Polynésie française; 3 Unité de Recherche sur les Maladies Infectieuses et Tropicales Emergentes, UM63, CNRS7278, IRD198, Inserm U1095, Centre national de référence des rickettsies, Coxiella et Bartonella. Institut Hospitalo-Universitaire Méditerranée-infection, Aix-Marseille université. Faculté de Médecine, 27 Bd jean Moulin, 13385 Marseille, cedex 5, France

**Keywords:** Rickettsia, Coxiella, Ehrlichia, Bartonella, Serosurvey, French Polynesia, Ticks, Cat fleas

## Abstract

**Abtract:**

**Background**

In the Pacific islands countries and territories, very little is known about the incidence of infectious diseases due to zoonotic pathogens. To our knowledge, human infections due to *Rickettsia* spp.*,* C*oxiella burnetii*, *Ehrlichia* spp. and *Bartonella* spp. have never been reported in French Polynesia; and infections due to *C. burnetti* have been reported worldwide except in New Zealand. To evaluate the prevalence of this disease, we conducted a serosurvey among French Polynesian blood donors.

**Methods**

The presence of immunoglobulin G antibodies against *R. felis*, *R. typhi*, *R. conorii*, *C. burnetii*, *B. henselae*, *B. quintana,* and *E. chaffeensis* was evaluated by indirect immunofluorescence assay in sera from 472 French Polynesian blood donors collected from 2011 to 2013. In addition, 178 ticks and 36 cat fleas collected in French Polynesia were also collected and tested by polymerase chain reaction to detect *Rickettsia* spp., *B. henselae* and *Ehrlichia* spp.

**Results**

None of the blood donors had antibodies at a significant level against *Rickettsia* spp.*,* C*oxiella burnetii*, *Ehrlichia* spp. and *Bartonella* spp. All tested ticks and cat fleas were PCR-negative for *Rickettsia* spp., *B. henselae*, and *Ehrlichia* spp.

**Conclusion**

We cannot conclude that these pathogens are absent in French Polynesia but, if present, their prevalence is probably very low. *C. burnetii* has been reported worldwide except in New Zealand. It may also be absent from French Polynesia.

## Background

French Polynesia (FP) is an Oceanian overseas territory of the French republic located in the south Pacific (274,000 inhabitants; 118 islands of which 67 are inhabited). It is one of the 22 Pacific Island Countries and Territories (PICTs) of the Oceania region. All PICTs conduct public health surveillance activities and submit their data to regional and international agencies. Laboratory facilities in PICTs include 20 level one laboratories (for screening tests) and 4 level two laboratories (for confirmation tests) [1]. When needed, specimens for specialized assays are referred to reference, or level 3, laboratories located in USA, Australia, New Zealand and France. Most often, frozen shipment of samples to level 3 laboratories is impossible. As few laboratory facilities are available in this region, little is known about the incidence and geographical distribution of infectious diseases due to neglected pathogens [2]. With the exception of leptospirosis known to be endemic in FP [3], there are few data available about the presence and incidence of bacterial zoonoses in this territory. In an effort to evaluate the seroprevalence against *Rickettsia* spp., agents of rickettsioses [4,5], *C. burnetii*, the agent of Q fever [6], *Bartonella* spp., agents of a variety of human and animal diseases [7] and *Ehrlichia* spp., agents of human erhlichioses [8], we conducted a serosurvey among 472 blood donors from Tahiti in collaboration with the French Polynesian blood bank centre (Papeete, FP) and the French Reference centre for Rickettsiosis (Marseille, France).

## Methods

A total of 472 blood donor samples collected from July 2011 to July 2013 were included in this study. None of them were collected specifically for the purpose of this study. Written consent was obtained by the blood bank centre from all blood donors before blood sampling. The study was approved by the Ethics Committee of the “Institut Fédératif de Recherche” 48, Aix-Marseille University, Marseille, France, under reference 13–021. A medical questionnaire was completed for all blood donors in accordance to the recommendations for blood donation policies, including the number of years of residency in FP. Serological tests were performed on samples collected for biological qualification of blood units. All samples were anonymized by the blood bank centre before testing for *Rickettsia* spp., *Bartonella* spp., *Ehrlichia* spp. *and Coxiella burnetii*. Four groups of blood donors were differentiated: natives from FP who never travelled abroad (group 1), natives from FP who travelled abroad for less than 1 week (group 2), natives from FP who travelled abroad for more than 1week (group 3), and immigrants (group 4). All study participants were healthy voluntary blood donors older than 18years old, and were all previously tested negative by the laboratory of the blood bank centre for HIV, HBV, HCV, HTLV, and *Treponema pallidum.*

The presence of immunoglobulin G class antibodies (IgG) against *Rickettsia. felis*, *R. typhi*, *R. conorii*, *C. burnetii* (total IgG), *Bartonella henselae*, *B. quintana,* and *Ehrlichia chaffeensis* was determined by indirect immunofluorescence assay (IFA) (the reference method for the serodiagnosis of infections caused by these bacteria), as previously described [9-13]. All sera were screened at a dilution of 1:50 and those positive at 1:50 were diluted and tested by IFA to determine end titers. One negative and one positive control (for each bacterium) were used for each set of 8 blood donors’ samples.

In addition, 178 ticks collected form dogs and cows and 36 fleas collected from dogs in 2012 in Tahiti, the main FP island, were identified according to standard taxonomic identification keys, and tested individually by polymerase chain reaction (PCR) to detect *Rickettsia* spp., *B. henselae* and *Ehrlichia* spp. as previously reported [14].

## Results

Numbers of blood donors in each group, age (mean, standard deviation and median) and number of years of residence in FP (mean, standard deviation and median) are reported in Table 1. All tested blood donors lived in Tahiti.

**Table 1:** Repartition of blood donors

	**Natives from FP**				**Immigrants**	**Total**
	**Group 1**	**Group 2**	**Group 3**	**Total natives**	**Group 4**	
Number of blood donors	102	215	23	340	132	472
Age						
Mean	32.1	34.9	34.5	34	42.6	36.5
Standard deviation	11.4	11.8	10.5	11.7	12.8	12.6
Median	31	32	31	32	42	35
Years of residence in FP						
Mean	32.1	34.9	30	33.7	15.7	28.8
Standard deviation	11.4	11.8	10.8	11.7	13	14.5
Median	31	32	29	32	12.5	28

IFA screening at the 1:50 dilution detected 23 positive sera, 9 for both *R. typhi* and *R. conorii*, 11 for *C. burnetii*, and 3 for both *B. quintana* and *B. henselae*. All positive sera were negative at the dilution of 1:100. According to the recommended IFA cutoff values for these diseases [6], all 472 blood donors were considered as non reactive against the tested antigens.

It should be noted that none of the 11 patients with sera positive at 1:50 for *C. burnetii* were from group 1 and 5 of them were from French immigrants living in French Polynesia. Only 2 of the 9 positive sera at 1:50 for both *R. typhi* and *R. conorii* were from group 1 and none of those positive at 1:50 for both *B. quintana* and *B. henselae* were from group 1.

Ticks were identified as *Rhipicephalus sanguineus* (132) or *Boophilus annulatus* (46), and fleas as *Ctenocephalides felis*. All ticks and cat fleas were tested negative by PCR for *Rickettsia* spp., *B. henselae* and *Ehrlichia* spp.

## Discussion

In PICTs, rickettsioses, Q fever, bartonelloses and ehrlichioses are not included in the list of nationally notifiable diseases. Therefore, their local epidemiology may only be extrapolated from investigations of outbreaks, serosurveys or public health and reference laboratory data.

Regarding rickettsioses, very few data are available from the whole Oceania region [15]. *R. felis*, the agent of flea-borne rickettsiosis, has been identified in Western Australia [16], New Zealand [17] and in New Caledonia [18]. Tick-borne rickettsioses have been reported in Australia: Queensland tick typhus (*R. australis*), Flinders Island spotted fever (*R. honei*) and variant Flinders Island spotted fever (*R. honei* strain “marmionii”) [19]. *Bartonella henselae* has been detected from New Caledonia children suffering from hepatic abscesses [20] and from cat fleas in New Zealand [17]. *Bartonella clarridgeiae*, suggested to be a minor etiologic agent of cat-scratch disease in humans has been isolated from cat fleas in FP [21], New Zealand [17] and New Caledonia [18].

Few serosurveys have been conducted in the Pacific region. In Queensland (Australia), a 5.6% seroprevalence of antibodies to at least one *Rickettsia* group (spotted fever, typhus and scrub typhus groups) was found among 920 human sera tested in 1996 [22]. In the same area, a 6.5% seroprevalence of antibodies to *C. burnetii* was detected among 447 sera collected in 2001 and 2002 [23].

Serosurveys conducted among blood donors were also informative: in southern France, a 18% seroprevalence of antibodies to *R. conorii* and 5% to *C. burnetii* was identified among 325 blood donors [24]; in 500 Tunisian blood donors, seroprevalences of antibodies to *R. conorii*, *R. typhi* and *C. burnetti* were found to be 9%, 3.6% and 26%, respectively [25]; in Spain, a 23.1% seroprevalence of antibodies to *C. burnetii* was found among 863 blood donors [26]; in 601 Turkish blood donors, a 32.3% seroprevalence of antibodies to *C. burnetii* was detected [27]; in Mauritania, 13.5, 19.6, 1.7 and 33% seroprevalences of antibodies to *R. conorii*, *R. africae*, *R. typhi* and *C. burnetti*, respectively, were observed among 118 blood donors and patients [28]; and in New Zealand, 5% of 140 blood donors had antibodies to *B. henselae*[29].

The blood donors included in this serosurvey study are representative of the French Polynesian population because 340 (72%) of them are natives from French Polynesia. Natives from French Polynesian are 87% according to the French Polynesian Institute of Statistics. They are also informative for past exposure to the studied bacteria because the mean duration of residency in FP was 33.7 years for native and 15.7 years for immigrant blood donors. Finally, based on the serosurveys detailed above, all of which detecting seroprevalences to *Rickettsia* spp., *Bartonella* and/or *C. burnetii* of 1.7 to 32.3% among populations of 118 to 601 blood donors, we believe that the 472 blood donor population that we tested is not too small to conclude that the seroprevalence of antibodies to the various tested pathogens is very low in French Polynesia.

The absence of antibodies to the investigated agents in FP may be explained by several factors. First, the arthropod vectors of rickettsiae and ehrlichiae may be absent in FP. To date, two tick species have been described in this territory, including *Rhipicephalus sanguineus* and *Boophilus annulatus*[30,31], the former being a known vector of spotted fever rickettsioses in other parts of the world. Our results confirmed that all 178 collected ticks were either *R. sanguineus* or *B. annulatus* ticks, but all were PCR-negative for *Rickettsia* spp., *B. henselae* and *Ehrlichia* spp. Therefore, currently, there is no proof that ticks may harbor human pathogens in FP. In addition, although *C. felis* fleas are proven vectors of *R. felis* and *B. henselae* in many areas worldwide [5], all 36 tested cat fleas in our study were PCR-negative. Thus, as *B. henselae* was not detected in cat fleas and cat-scratch disease is not diagnosed in children in FP, we assume that cats in this territory are not infected. Few numbers of ruminants (goats, sheep, cattle), the reservoirs of *C. burnetii*, are present in FP. In addition, FP has very strict importation criteria for animals and plants. For pets and ruminants notably, a quarantine period and a non contagiosity certificate are mandatory before introduction of the animal. In 1996, a Q fever serosurvey conducted on 306 farms mammals (sheep and goats) by the veterinary department of the French Polynesian Ministry of Health detected no positive. Therefore, we believe that Q fever is absent from FP*.* Furthermore, as the disease is also absent from New Zealand [32], we assume that it may be absent from most of the Pacific Ocean area.

Serodiagnostic tests for these bacteria are occasionnally performed for FP patients but, to date, results have always been negative. This means that a positive serology against *Rickettsia* spp.*, C. burnetti*, *Ehrlichia* spp. or *Bartonella* spp. should be interpreted with caution. It is of particular importance because all of these pathogens are able to cause unspecific febrile illness associated with thrombocytopenia, leukopenia, and increased serum transaminases, which is a common presentation of dengue, a major public health problem in FP [33].

## Conclusions

From our study, we cannot prove that rickettsioses, ehrlichioses, bartonelloses and Q fever are absent in FP but we can consider that, if present, their prevalence is very low. Further testing of patients with unexplained fever, will be conducted to confirm the absence of rickettsoses in this area.

## Abbreviations

FP: French Polynesia; PICTs: Pacific Island Countries and Territories; IgG: Immunoglobulin G class antibodies; IFA: indirect immunofluorescence assay.

## Competing interests

The authors declare that they have no competing interests.

## Authors’ contributions

MD: FG BJ: ES PP: FG RD: FG FPE: JY FG. All authors read and approved the final manuscript.

## Authors’ information

D.M. is medical doctor, director or the diagnosis laboratory and of the “unit of emerging infectious diseases” of the “Louis Malarde Intitute”, a public health organism in charge for investigations in the field of infectious diseases in French Polynesia. J.B. is medical doctor, director of the French Polynesian blood Bank. P.P. is Professor of Infectious Diseases and Tropical Medicine at the Medicine School of Marseille, France. He is Director of the WHO Collaborative Center (FRA 75) for Rickettsial and other Arthropod Borne Bacterial Diseases. D.R. is Professor of Microbiology at the Medicine School of Marseille, France. He is director of the “research unit on infectious and tropical emerging diseases” in Marseille, France. He is co-author of more than 1,000 publications in the field of infectious diseases. P.E.F. is Professor of microbiology at the Medicine School and at the “research unit on infectious and tropical emerging diseases” in Marseille, France. He is Director of the French reference center for Rickettsia, Coxiella and Bartonella.

## Pre-publication history

The pre-publication history for this paper can be accessed here:

http://www.biomedcentral.com/1471-2334/14/255/prepub
